# Carboxymethyl Dextran-Based Nanomicelle Coatings on Microarc Oxidized Titanium Surface for Percutaneous Implants: Drug Release, Antibacterial Properties, and Biocompatibility

**DOI:** 10.1155/2022/9225647

**Published:** 2022-07-12

**Authors:** Weiliang Ye, Minghao Zhou, Luxuan Zhang, Jingwei Yu, Junjun Fan, Hongbo Wei

**Affiliations:** ^1^Department of Pharmaceutics, School of Pharmacy, Fourth Military Medical University, Xi'an 710032, China; ^2^State Key Laboratory of Military Stomatology, National Clinical Research Center for Oral Diseases & Shaanxi Engineering Research Center for Dental Materials and Advanced Manufacture, Department of Oral Implants, School of Stomatology, The Fourth Military Medical University, Xi'an 710032, China; ^3^Department of Orthopedics, The First Affiliated Hospital of Air Force Military Medical University, Xi'an 710032, China

## Abstract

Bacterial contamination and biofilm formation onpercutaneous implants can lead to device failure and be life-threatening. To solve this issue, we constructed a carboxymethyl dextran- (CMD-) based nanomicelle antibacterial coating on the microarc-oxidized titanium (MAO-Ti) surface (described in the supplementary file). The self-assembled CMD-based nanomicelles and octadecylamine (ODA) were developed as a drug carrier and loaded with minocycline (MC). The characterization and stability of the MC-loaded nanomicelles were determined. The surface roughness, hydrophilicity, and drug release property of the coatings were also investigated. Our findings showed that the cross-linked MC-loaded nanomicelles (MC@(ODA-CMD)_CL_) were more stable than the uncross-linked nanomicelles. Moreover, MC@(ODA-CMD)_CL_ was successfully incorporated into the pores of MAO-Ti, which significantly increased the surface hydrophilicity of the coatings without influencing their surface roughness. In addition, the coatings demonstrated a sustained release time of 360 h, with a cumulative release rate reaching 86.6%. *Staphylococcus aureus* (S. aureus) was used to determine the antibacterial properties of the coatings, and human skin fibroblasts were seeded on them to investigate their biocompatibility. The results showed that the coatings significantly reduced the number of adhesive S. aureus and promoted the viability, adhesion, and morphology of the human skin fibroblasts compared to smooth titanium (S-Ti) sheets. In conclusion, MC-loaded CMD-based nanomicelles coated on MAO-Ti surface (MC@(ODA-CMD)_CL_-Ti) demonstrated sustained-release properties, excellent antibacterial properties and biocompatibility, and promising potential as coatings for percutaneous implants.

## 1. Introduction

For patients with congenital or acquired craniomaxillofacial defects, implant-retained prostheses can restore their organ structure and functions to improve their quality of life [1–3]. However, it was found that 27.3% of patients with a history of radiotherapy and 6.6% of patients without a history of radiotherapy experienced percutaneous implant failure within three years [4]. Meanwhile, up to 72.3% of patients develop inflammation around the percutaneous implant [5]. These often cause device failure, forcing surgeons to replace or remove them and resulting in psychological, physical, and financial challenges to the patients [5, 6].

In previous studies, the long-term effectiveness of a percutaneous implant was shown to not only depend on implant osseointegration but also on soft tissue sealing since this also serves as a biological barrier protecting the implant from the outside environment [7, 8]. The main initiator leading to percutaneous implant failure is bacterial invasion, as they can break the seal between the skin and the implant [9, 10]. The penetration of microbes during the surgical healing process disrupts soft tissue attachment to the percutaneous portion of the implant and the formation of stable skin-implant sealing, leading to inflammatory peri-implant diseases [11, 12]. In fact, the relation between soft tissue integration and microbial adhesion can be conceptualized as a “race for the surface,” whereby soft tissue cells and microbes compete to occupy the implant's surface [13, 14]. If microbes become the dominant entity at the surface of the implant, this decreases the likelihood of host cells to establish robust and stable soft tissue integration around the implants. To solve this issue, functionalized percutaneous devices with antibacterial ability could be used as an effective way to improve the success rate of percutaneous implants.

Compared with systemic antibacterial administration, the preparation of antibacterial coatings on implants' surfaces can achieve a much higher local drug concentration around the implants, avoiding systemic toxicity and preventing bacterial drug resistance [15, 16]. Therefore, implant surface coating designs such as nanosilver-loaded electrostatic self-assembly composite coating [17], antibacterial peptide sustained-release coating [18], and multilayer modified plasma-sprayed porous coating [19] have been widely investigated to endow implants with antibacterial properties. Our team has developed an antibiotic nanodelivery coating on MAO titanium based on self-decomposable silica-gentamycin nanoparticles, which showed excellent antibacterial effects [20]. However, they are often associated with limitations such as instability of drug carriers, insufficient drug release time, and potential cytotoxicity.

Comparatively, nanomicelles are more suitable drug carriers to create an antibacterial coating for implants. They have a relatively smaller size, higher drug loading ability, and longer sustained release time and have already been applied in various biological and medical fields [21–23]. Nanomicelles are nanosized colloidal carriers characterized by core-shell structures with an exterior hydrophilic shell and interior hydrophobic core. When the critical micelle concentration (CMC) is reached, amphiphilic molecules bury the hydrophobic drugs within their hydrophobic core while they self-assemble [24]. Dextran and its derivatives are biopolymers that have many advantages over synthetic polymers, such as excellent solubility in water, biocompatibility, and nontoxicity for constructing nanomicelles for drug delivery [25, 26]. Moreover, nanomicelles based on dextran and its derivatives have been shown to promote drug dissolution and improve drug bioavailability [27]. Carboxymethyl dextran (CMD) contains carboxyl groups at its terminal for chemical binding. It can be conjugated with octadecylamine (ODA), a primary alkylamine commonly used as a hydrophobic surface modifier [28]. Thus, CMD and ODA can serve as hydrophilic and hydrophobic segments as they bind together via amide bonds to constitute amphiphilic block copolymers used to form self-assembled nanomicelles in the preparation process.

MC has an apparent antibacterial effect on implant-associated infections and is widely used in conditions such as periodontitis and peri-implantitis. It can reversibly bind to the helical region (H34) of the 30S ribosome, preventing amino acid residues from being incorporated into the extended peptide chain, resulting in loss of peptide formation and bacterial growth [29]. S. aureus has been found to be the most common bacteria in percutaneous implant-related infections. It can adhere to the implant surface at an early stage and promote the accumulation of other bacterias, resulting in biofilm formation [16, 30].

In this study, we chose MC as model antibiotics to investigate their antibacterial property against S. aureus on the coatings of implants. We first prepared self-assembled nanomicelles based on CMD and ODA. Then, the dialysis method was used to encapsulate MC into the hydrophobic core of the nanomicelles. Lastly, we constructed shell cross-linked MC-loaded nanomicelles on the microarc-oxidized titanium surface (MC@(ODA-CMD)_CL_-Ti). To evaluate the applicability of the model, we compared the characteristics of shell cross-linked drug-loaded nanomicelles (MC@(ODA-CMD)_CL_) with uncross-linked ones (MC@ODA-CMD). Further, we also analyzed the drug release performance, antibacterial properties, and the possible influence of MC-loaded CMD-based nanomicelle coatings on human skin fibroblasts.

## 2. Materials and Methods

### 2.1. Synthesis and Characterization of ODA-CMD

**Figure 1:**
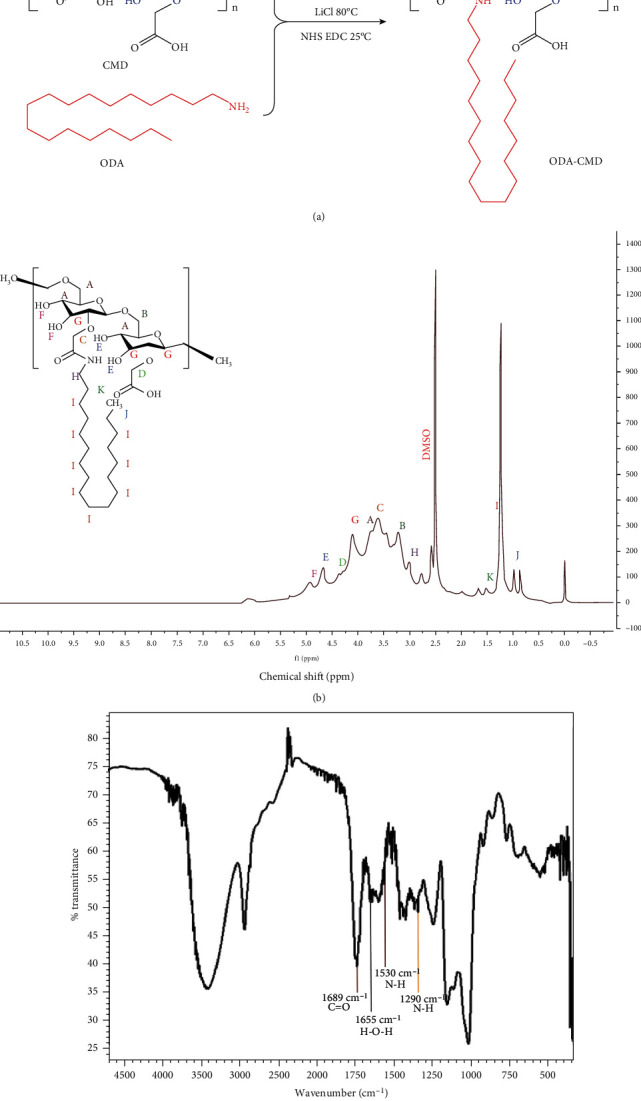
Characterization and biocompatibility of nanomicelles. (a) The synthesis route of ODA-CMD. (b) ^1^H NMR spectra of ODA-CMD. (c) Infrared spectra of ODA-CMD.

ODA-CMD was prepared as shown in Figure 1(a). First, 0.6 g of CMD (carboxyl equivalent to 1 mmol; J&K Scientific Ltd., Beijing, China) and lithium chloride (LICI; 21.3 mg and 0.5 mmol; Sigma-Aldrich, St. Louis, USA) was added to 5 mL of dimethylformamide (DMF) in a round-bottom flask at 80°C and stirred until dissolved completely. Then, 1-ethyl-3-(3-dimethylaminopropyl) carbodiimide hydrochloride (EDC; 287.6 mg and 1.5 mmol; J&K Scientific Ltd., Beijing, China) and N-hydroxysuccinimide (NHS; 172.5 mg and 1.5 mmol; J&K Scientific Ltd., Beijing, China) were added to the reaction, which was then stirred at room temperature for about 1 h.

Next, octadecylamine (ODA; 54 mg and 0.2 mmol; J&K Scientific Ltd., Beijing, China) was dissolved into 2 mL of DMF. The mixture was then slowly dropped to the above reaction for 24 h at room temperature using a dropping funnel. Finally, the reaction mixture was precipitated by adding ethanol and purified by repeated washing with methanol three times. The obtained product was dialyzed using cellulose dialysis membranes (MWCO: 1.5 kDa) against deionized water and lyophilized to obtain ODA-CMD. The ODA-CMD was confirmed by 1H NMR spectra recorded on a 300 MHz (Varian Mercury Plus 300, Palo Alto, CA, USA) nuclear magnetic resonance instrument, using DMSO-d6 as solvent and tetramethylsilane (TMS) as the internal reference. The structure of ODA-CMD was further characterized by FTIR spectrarecorded on KBr pellets (FTIR-8400S spectrometer, Shimadzu, Kyoto, Japan) from 400 cm^−1^ to 4000 cm^−1^.

### 2.2. Preparation and Characterization of Drug-Loaded Nanomicelles

#### 2.2.1. Preparation of Drug-Loaded Nanomicelles

First, 4 mg of minocycline hydrochloride (MC·HCl; J&K Scientific Ltd., Beijing, China) was added to 4 mL of dimethyl sulfoxide (DMSO). Then, 4 *μ*L of triethylamine (TEA) was added to the resulting solution. After the dissolution was complete, they were heated and stirred in an oil bath at 60°C, away from light exposure. Next, 10 mg of polymer materials ODA-CMD was added to the reaction solution. After stirring the mixture for 2 h, 16 mL of ethylenediamine was slowly added (dropwise) and stirred for another 12 h. The mixture was packed into a dialysis bag (interception molecular weight: 2000 Da) and dialyzed in water for 48 h. The water was changed every 6 h. Shell cross-linked drug-loaded nanomicelles (MC@(ODA-CMD)_CL_) were obtained by freeze-drying after dialysis. In addition, uncross-linked drug-loaded nanomicelles (MC@ODA-CMD) were prepared from distilled water without ethylenediamine as the control.

#### 2.2.2. Encapsulation Efficiency and Drug-Loading Capacity

To determine the drug loading and entrapment efficiency, the freeze-dried drug-loaded micelles were dissolved in 3 mL DMSO and incubated in a water bath at 40°C for 3 h. The concentration of MC was measured at 352 nm using an ultraviolet spectrophotometer (UV-2001 Spectrophotometer, Shimadzu, Japan). The measurement was performed in triplicate. Drug loading and entrapment efficiency were calculated using the following equations:

(1) $$\begin{array}{c} \text{DL}\left(\%\right)=\frac{\text{Weight of MC in the micelles}}{\text{Weight of the micelles}}\times 100\%, \end{array}$$

(2) $$\begin{array}{c} \text{EE}\left(\%\right)=\frac{\text{Weight of MC in the micelles }}{\text{Weight of the feeding MC}}\times 100\%. \end{array}$$

#### 2.2.3. Particle Size and Zeta Potential

An adequate amount of drug-loaded micelles was dissolved in distilled water, and the micellar solution, at a concentration of about 1 mg/mL, was filtered by a 0.22 *μ*m filter membrane. Particle size and zeta potential were measured using a nanoparticle size analyzer and zeta potential analyzer. The experiment was performed three times, and the average value was used.

#### 2.2.4. Morphology of Drug-Loaded Nanomicelles

The morphology of the micelles was observed via transmission electron microscopy (TEM; JEOL-100CXII, Japan). The micellar solution, at a concentration of 0.5 mg/mL, was prepared, filtered with a 0.22 *μ*m filter membrane, and dripped on the copper omentum on the micelles. When the solvent was almost dry, a drop of phosphotungstic acid (Sigma-Aldrich, St. Louis, USA) solution was added for negative staining and observed with TEM after drying.

#### 2.2.5. Stability of Drug-Loaded Nanomicelles

Briefly, 2.5 mg drug-loaded micelles were dispersed in 2.5 mL phosphate buffer solution (pH 7.4) and stored in a 37°C water bath in the dark for 1, 5, 10, 15, 20, 25, and 30 days, respectively. Then, the particle size was measured. Their stability was evaluated by observing the particle size and the polymer dispersity index (PDI) under dynamic light scattering (DLS; Beckman Coulter Particle Analyzer, Fullerton, CA, USA). Then, 1 mg/mL of drug-loaded micelles was dissolved in pH 7.4 PBS and stored in a water bath at 37°C in the dark for 1 day and 30 days, respectively. TEM was used to assess their morphological changes and evaluate their stability.

#### 2.2.6. Determination of CMC

The value of CMC is an important index of micelle stability, which can be measured using the pyrene fluorescence probe method. Here, 0.1 *μ*mol/L of pyrene stock solution was prepared with acetone, and 100 mg ODA-CMD was dissolved in 10 mL of distilled water to obtain a 10 mg/mL stock solution. The stock solution was then diluted to 10, 5, 1, 0.5, 0.1, 0.05, 0.01, 0.005, 0.001, 0.0005, 0.0001, 0.00005, and 0.00001 mg/mL, respectively, to which 50 *μ*L of pyrene solution was added. They were then incubated in a water bath at 37°C for 6 h. After acetone was completely volatilized, the fluorescence intensities I394 and I373 were detected using a fluorescence spectrophotometer (Shanghai Precision and Scientific Instrument Co. Ltd., Shanghai, China) at an excitation wavelength of 339 nm and an emission wavelength of 373 and 394 nm. The curve was developed using concentration as the vertical coordinates and I394/I373 as the horizontal coordinates. CMC was used as the inflection point. The CMC measurement of (ODA-CMD)_CL_ was the same as above.

### 2.3. Evaluation of the Biocompatibility of Nanomicelles

#### 2.3.1. Hemolysis Assay

Here, 8 mL of rabbit's blood was added to a certain amount of heparin (Sigma-Aldrich, St. Louis, USA) for anticoagulation and diluted with 10 mL of normal saline. Then, 100 *μ*L of the diluted blood was added to different concentrations of 1 mL micellar solution. After a warm bath at 37°C for a certain period, the mixture was centrifuged at 750 g for 5 min. The supernatant was added to a 2 mL of ethanol/hydrochloric acid mixture (39 : 1; 99% ethanol (*v*/*v*): 37% hydrochloric acid (*w*/*v*)), and after centrifugation for another 5 min, the supernatant was taken. The absorbance was then measured at 398 nm with an ultraviolet spectrophotometer (TU-1901, Beijing Purkinje Co., China). Distilled water was used as the positive control and 0.9% of normal saline as the negative control, all of which were treated with the same method. The hemolysis rate of the micelle solution (HR%) was calculated using the following formula:

(3) $$\begin{array}{c} \text{HR }\left(\%\right)=\frac{A_{\text{test}}-A_{\text{negative}}}{A_{\text{positive}}-A_{\text{negative}}}\times 100\%, \end{array}$$

where “*A*_test_” refers to the absorbance of the experiment tube, “*A*_negative_” refers to the absorbance of the negative control tube, and “*A*_positive_” refers to the absorbance of the positive control tube.

#### 2.3.2. Cytotoxicity Assay

The safety of the micellar materials was evaluated by evaluating the toxic effect of the micellar materials on human umbilical vein endothelial cells (HUVECs) (Institute of Biochemistry and Cell Biology, Chinese Academy of Science, Shanghai, China). The HUVECs were suspended in RPMI-1640 (HyClone, UT, USA) medium containing 10% fetal bovine serum (HyClone, UT, USA) and incubated at 37°C with 5% CO_2_ in an incubator. Logarithmic growth phase cells were inoculated in a 96-well culture plate and cultured overnight in a CO_2_ incubator. After a pipettor was used to suction the culture medium, different concentrations of blank micelle solution were added and incubated for 24 h. Then, 20 *μ*L of 3-(4,5-dimethylthiazol-2-yl)-2,5-diphenyltetrazolium bromide (MTT; J&K Scientific Ltd., Beijing, China) solution (5 mg/mL) was added and incubated for 4 h, after which the culture medium was discarded, and 200 *μ*L DMSO was added. The absorbance was measured at 490 nm using a microplate reader (Bio-Rad Laboratories, Inc., CA, USA). The survival rates of the HUVECs were calculated using the following equation:

(4) $$\begin{array}{c} \text{Survival rate}=\frac{\text{Absorbance value after administration}}{\text{Absorbance value of negative control}}\times 100\%. \end{array}$$

The corresponding survival graph was built using drug concentration as the horizontal ordinate and survival rates as the vertical ordinate.

### 2.4. Fabrication and Characterization of Titanium Specimens (S-Ti, MAO-Ti, MC@(ODA-CMD)_CL_-Ti)

Commercial pure titanium sheets (Northwest Institute for Nonferrous Metal Research, Xi'an, China) in disk forms (10 mm in diameter and 2 mm in thickness) were mechanically ground using a 400-7,000 grit silicon carbide paper and cleaned ultrasonically in acetone (FUYU Fine Chemicals Co., Ltd.), ethanol, and deionized water.

The MAO procedure was performed in a mixed electrolyte containing 0.04 M *β*-glycerophosphate sodium (Ruixi Biological Technology Co., Ltd., Xi'an, China) and 0.2 M calcium acetate (Ruixi Biological Technology Co., Ltd.) using a pulsed direct current power supply. The applied voltage, oxidizing time, frequency, and duty cycle were 300 V, 5 min, 600 Hz, and 8.0%, respectively. Afterward, the surfaces of the specimens were rinsed with deionized water and air-dried.

The drug-loaded micelles with 600 *μ*g of MC were added into 1 mL of 0.2% gelatin solution. Ultrasonic treatment was given for 10 min to form a suspension, which was then added dropwise to evenly lay on the surface of the titanium sheet with a diameter of 1 cm, shaken for 1 h, and dried. The titanium surface was washed with ethanol three times and freeze-dried.

The surface morphology of S-Ti, MAO-Ti, and MC@(ODA-CMD)_CL_-Ti was observed by scanning electron microscope (SEM, S-4800; Hitachi High-Technologies, Tokyo, Japan).

### 2.5. Characterization of Modified Titanium Specimens

#### 2.5.1. Contact Angle

Contact angle measurements were carried out to examine the surface hydrophilicity of the titanium specimens (S-Ti, MAO-Ti, and MC@(ODA-CMD)_CL_-Ti) using the sessile drop measurement method and the EasyDrop Standard instrument (KRÜSS GmbH, Hamburg, Germany) at ambient temperature. Two different liquids (ultrapure water and formamide) were used. The experiments were performed in triplicate for each type of specimen.

#### 2.5.2. Roughness

The surface morphology and roughness of the titanium specimens (S-Ti, MAO-Ti, and MC@(ODA-CMD)_CL_-Ti) were analyzed by atomic force microscopy (Veeco Germany, Mannheim, Germany). Their relative surface area (Ra) and root mean square (RMS) roughness were measured to distinguish the roughness features of the different titanium surfaces. Six tests were performed on randomly selected areas, and the average values were recorded.

### 2.6. *In Vitro* Release Test of Drug-Loaded Nanomicelle Coating on Titanium Surface

PBS at pH 7.4 was used as the release medium in this study. The drug release experiment was carried out using the dialysis bag method. First, 2 mL of a mixture containing an equal dose of Ti loaded with free MC (MC-Ti), MC@(ODA-CMD), MC@(ODA-CMD)_CL_-Ti was loaded into the dialysis bags (3500 g/mol), placed in an Eppendorf tube containing 5 mL of release medium, and shaken in a constant temperature oscillator (37°C and 60 rpm). The samples were taken at indicated preset time points (1 h, 12 h, 24 h, 3 d, 5 d, 9 d, 11 d, and 15 d), and all release media in the release tube were replaced with fresh media to ensure that the MC leak condition of minocycline was met. The drug content in the sample was determined by ultraviolet spectrophotometry, and the cumulative release percentage of the drug was calculated.

### 2.7. Evaluation of Antibacterial Properties

#### 2.7.1. Antibacterial Rate (AR)

*Staphylococcus aureus* (*S. aureus*) (ATCC 25923; ATCC, Manassas, VA, USA) was cultured in a Mueller-Hinton medium (Aobox Biotechnology, Beijing, People's Republic of China) until the midlogarithmic period. The bacterial solution was adjusted to a concentration of 10^6^ CFU/mL. The specimens (S-Ti, MAO-Ti, and MC@ (ODA-CMD)_CL_-Ti) were individually incubated in 1 mL of the bacterial suspension at 37°C for 24 h. Each titanium specimen was then rinsed twice with PBS. The bacteria attached to the specimens were isolated with a 5 mL PBS solution for 5 minutes. Then, the bacterial suspensions were recultivated in Mueller-Hinton agar (MHA) plates for colony counting. The AR was calculated using the following equation:

(5) $$\begin{array}{c} \text{AR}\left(\%\right)=\frac{\text{CFUcontrol}-\text{CFUexperiment}}{\text{CFUcontrol}}\times 100\%. \end{array}$$

Here, S-Ti served as the control group and MAO-Ti and MC@ (ODA-CMD)_CL_-Ti as the experimental groups.

#### 2.7.2. Bacterial Viability and Morphology

The specimens (S-Ti, MAO-Ti, and MC@(ODA-CMD)_CL_-Ti) were incubated in 1 mL bacterial suspension (10^6^ CFU/mL) for 24 h and then rinsed twice with PBS.

*(1) Bacterial Viability*. The specimens were stained with SYTO-9 and propidium iodide (PI) and dyed (LIVE/DEAD BacLight^TM^ Bacterial Viability Kits, L13152; Life Technologies Corp., Carlsbad, CA, USA) for 15 min without light exposure. Live bacteria (intact membranes) were stained with SYTO-9 (green), and dead bacteria (damaged membranes) were stained with PI (red), followed by examination using laser scanning confocal microscopy (FV1000; Olympus, Tokyo, Japan). The percentage of dead bacteria was calculated as the ratio of red to total fluorescence using the ImageJ software according to the following equation:

(6) $$\begin{array}{c} \text{Red fluorescence ratio }\left(\%\right)=\frac{\text{Red fluorescence intensity}}{\text{Red and green fluorescence intensity}}\times 100\%. \end{array}$$

*(2) Bacterial Morphology*. The specimens were fixed with 3% glutaraldehyde at 4°C overnight and dehydrated in a series of ethanol solutions, each for 8 minutes. The specimens were dried and gold-sputtered before observation under SEM.

#### 2.7.3. CCK-8 Assay

CCK-8 assay was used to examine the antibacterial performance of coated Ti specimens [31]. Briefly, the S. aureus culture was diluted in a 1 : 5 ratio, and the bacterial suspension (190 *μ*L) was added to each titanium specimen in a culture medium. After 24 h of incubation, the Ti specimens were removed with a fresh culture medium, to which a BHI medium was added. The culture medium was sealed and treated with ultrasound for 5 min at 20°C. Then, 10 *μ*L of CCK-8 reagent was added, and their OD values (600 nm) were measured.

### 2.8. Behavior of Fibroblasts on Titanium Specimens

#### 2.8.1. Cell Cultivation

Primary human skin fibroblasts were harvested and cultured as previously described [32]. Cells were cultured in DMEM (HyClone, UT, USA) medium containing 10% fetal bovine serum (HyClone, UT, USA) in an incubator with 5% CO_2_/95% air and humidified condition at 37°C. The cells were grown, and passages 3-8 were collected and seeded onto the titanium specimens (S-Ti, MAO-Ti, and MC@(ODA-CMD)_CL_-Ti). The specimens were then placed in a 48-well plate and cultivated in a CO_2_ incubator, with the cell culture media changed every 2 days.

#### 2.8.2. Cell Viability

Fibroblasts at a concentration of 1000 cells/cm^2^ per specimen substrate were added to a standard 48-well plate. Cell viability assay was performed using a Cell Counting Kit (Dojindo Molecular Technologies, Kumamoto, Japan) following the manufacturer's instructions. Briefly, after 1, 3, 5, and 7 days of incubation, 20 *μ*L of CCK-8 solution and 200 mL of fresh culture medium were added to each well for 2 h at 37°C with 5% CO_2_. The absorbance was measured at 490 nm. The experiment was performed with a sample size of *n* = 6.

#### 2.8.3. Cell Adhesion

Cell adhesion assay was conducted using a living cell labeling kit (Cellstein-CFSE; Dojindo). Briefly, carboxyfluorescein diacetate succinimidyl ester was used to label the fibroblasts (10^7^ cells/mL) for 15 min at 37°C. Then, the cells were pelleted by centrifugation, and the pellets were resuspended in a fresh medium to wash the cells. This washing process was repeated thrice. Following this, the fibroblasts were inoculated onto standard 48-well culture plates at a density of 250,000 cells per substrate, and the adhesion experiment was performed under standard cell conditions. The substrates were rinsed with PBS to remove unadhered cells, and the fluorescent dye was dissolved by adding 200 mL sodium dodecyl sulfate/Tris (hydroxymethyl) aminomethane solution to each well. An aliquot of 150 mL was taken from each well and transferred to a fresh 96-well plate. Fluorescence intensity was measured using a Synergy HT reader (BioTek, Winooski, VT, USA). All experiments were performed with a sample size of *n* = 6.

#### 2.8.4. Acridine Orange/Ethidium Bromide (AO/EB) Staining

Titanium specimens were seeded with 1 × 10^5^ fibroblasts per well in 12-well plates for 24 h. The fibroblasts in each well were rinsed with PBS and stained with 2 mL of AO/EB reagent (Sigma-Aldrich, USA) at 27°C for 10 min after removing the supernatant. Subsequently, the cells were rinsed twice with PBS and observed under an inverted fluorescence microscope.

### 2.9. Statistical Analysis

All experiments were repeated in parallel, at least three times, and the data are presented as mean ± standard deviation. Statistical analysis was performed using the Prism 9.2.0 (GraphPad Software, La Jolla, CA, USA) software with one-way or two-way analysis of variance (ANOVA). Statistical significance was considered for *P* values < 0.05.

## 3. Results

### 3.1. Characterization of ODA-CMD

ODA-CMD was found to turn to a white loose solid after freeze-drying and was identified by proton nuclear magnetic resonance (^1^HNMR). The chemical shift of protons on the glucoside ring of glucan that appeared at 3.15-3.85 ppm (Figure 1(b), A–C) and 4.25-5.12 ppm (Figure 1(b), D–F) was the chemical shift of the hydroxyl protons on the glucan and the protons on the heterotopic carbon, respectively. The 0.85 ppm (Figure 1(b), J) and 1.24-1.5 (Figure 1(b), I and K) ppm can be referred to as the end methyl of the octadecylamine and intermediate methylene proton peaks, respectively. The chemical shift observed at 4.12 ppm (Figure 1(b), G) was the upper methylene proton peak after carboxymethylation of dextran. The 3.09 ppm (Figure 1(b), H) was assigned to the proton peak of methylene closest to the amide bond.

Polymer materials were identified using the Fourier transform infrared (FTIR) spectroscopy. As shown in Figure 1(c), the characteristic peaks at 1689 cm^−1^ came from the stretching vibration of the amide bond C=O, 1530 cm^−1^ came from the stretching vibration of the amide bond N-H, and 1290 cm^−1^ came from the stretching vibration of the amide bond C-N. The absorption peak of 1655 cm^−1^ was produced by the hydrogen bond H-O-H of the dextran. The above results confirmed that the synthetic polymer ODA-CMD was the target material.

### 3.2. Characterization of Drug-Loaded Nanomicelles

#### 3.2.1. Biocompatibility of Blank Nanomicelles

**Figure 2:**
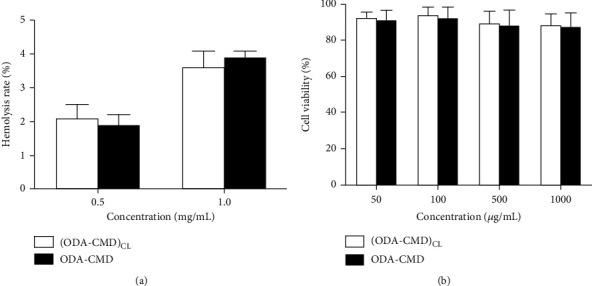
(a) Hemolysis rate of rabbit RBCs with two types of blank nanomicelles at different concentrations. The data are presented as mean ± SD (*n* = 3). (b) Cell viability in HUVECs after treatment with two kinds of blank micelles for 24 h. Data are presented as mean ± SD (*n* = 3).

Although the hemolysis rate is concentration-dependent, the hemolysis rates of two kinds of blank nanomicelles were found to be less than 5%, even at high concentrations. No significant difference was observed between ODA-CMD and (ODA-CMD)_CL_ (Figure 2(a)). These results indicated that the two types of blank nanomicelle materials did no damage to RBC when the concentration was less than 1.0 mg/mL. Further, the viability of HUVECs treated with different concentrations of ODA-CMD and (ODA-CMD)_CL_ did not change significantly, and the overall viability rate of HUVECs was above 90% (Figure 2(b)). These results showed that the two kinds of micellar materials had no toxic effect on HUVECs.

#### 3.2.2. Characteristics of Drug-Loaded Nanomicelles

**Figure 3:**
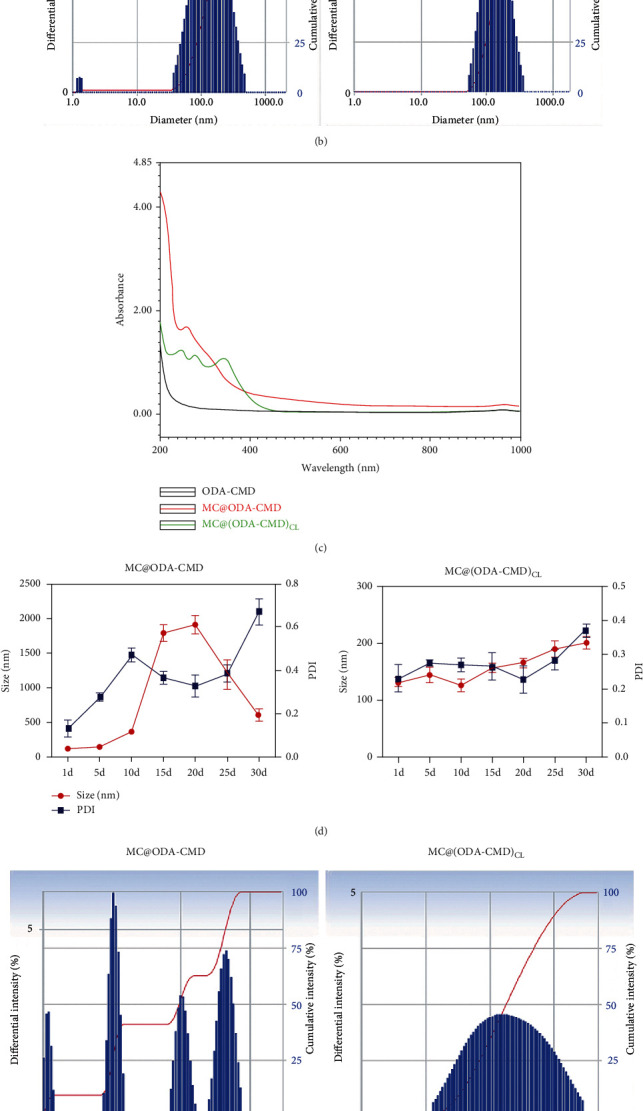
Characterization and stability of drug-loaded nanomicelles. (a) TEM images of MC@ODA-CMD and MC@(ODA-CMD)_CL_. (b) DLS analysis of MC@ODA-CMD and MC@(ODA-CMD)_CL_. (c) Ultraviolet spectra of MC@ODA-CMD and MC@(ODA-CMD)_CL_. (d) Changes in particle size of MC@ODA-CMD and MC@(ODA-CMD)_CL_ after incubation in PBS buffer for 30 days. (e) DLS results of particle size of MC@ODA-CMD and MC@(ODA-CMD)_CL_ after incubation in PBS buffer for 30 days. (f) TEM images of MC@ODA-CMD and MC@(ODA-CMD)_CL_ after incubation in PBS buffer for 30 days.

As shown in Table 1, the approximate particle size of MC@(ODA-CMD)_CL_ was 130 nm, and that of MC@ODA-CMD was 118 nm. The PDI value indicated that the micelles had uniform particle size distribution. The zeta potentials of the two types of micelles were negative, with an absolute value greater than 10 mV, which could effectively avoid the aggregation of the micelles and maintain their stability. The drug loading and encapsulation rate of MC@(ODA-CMD)_CL_ were slightly higher than those of MC@ODA-CMD. In addition, the CMC value of MC@(ODA-CMD)_CL_ was lower than that of MC@ODA-CMD, and the lower CMC value indicated that the cross-linked micelle MC@(ODA-CMD)_CL_ possessed better stability. TEM showed that MC@(ODA-CMD)_CL_ and MC@ODA-CMD were spherical and well dispersed (Figure 3(a)). The particle size distributions of MC@(ODA-CMD)_CL_ and MC@ODA-CMD were determined by DLS (Figure 3(b)). The scanning results of the ultraviolet spectrophotometer showed that MC@(ODA-CMD)_CL_ and MC@ODA-CMD had the characteristic absorption peak of MC, while the blank material had no ultraviolet absorption (Figure 3(c)). These results further indicated that the nanomicelles successfully encapsulated MC without affecting its structure.

#### 3.2.3. Stability of Drug-Loaded Nanomicelles

The changes in particle size and PDI of MC@(ODA-CMD)_CL_ and MC@ODA-CMD incubated in PBS buffer at pH 7.4 for 30 days were investigated by DLS (Figures 3(d) and 3(e)). The particle size and PDI of MC@ODA-CMD were observed to suddenly increase after 10 days, while the particle size and PDI of MC@(ODA-CMD)_CL_ did not change significantly within the 30 days. The morphological changes of MC@(ODA-CMD)_CL_ and MC@ODA-CMD incubated in PBS buffer at pH 7.4 for 30 days were observed using TEM. The size of MC@(ODA-CMD_)CL_ was observed to slightly increase after incubation in the pH 7.4 buffer for 30 days, with the micellar morphology still maintained. After incubation in the buffer for 30 days, MC@ODA-CMD was found to dissolve entirely, and its micellar morphology could not be observed (Figure 3(f)). The above results indicated that the stability of nanomicelles was significantly improved after cross-linking, laying a foundation for its long-term drug release.

### 3.3. Characterization of Modified Titanium Specimens

#### 3.3.1. Observation of the Surfaces of Titanium Specimens by SEM

**Figure 4:**
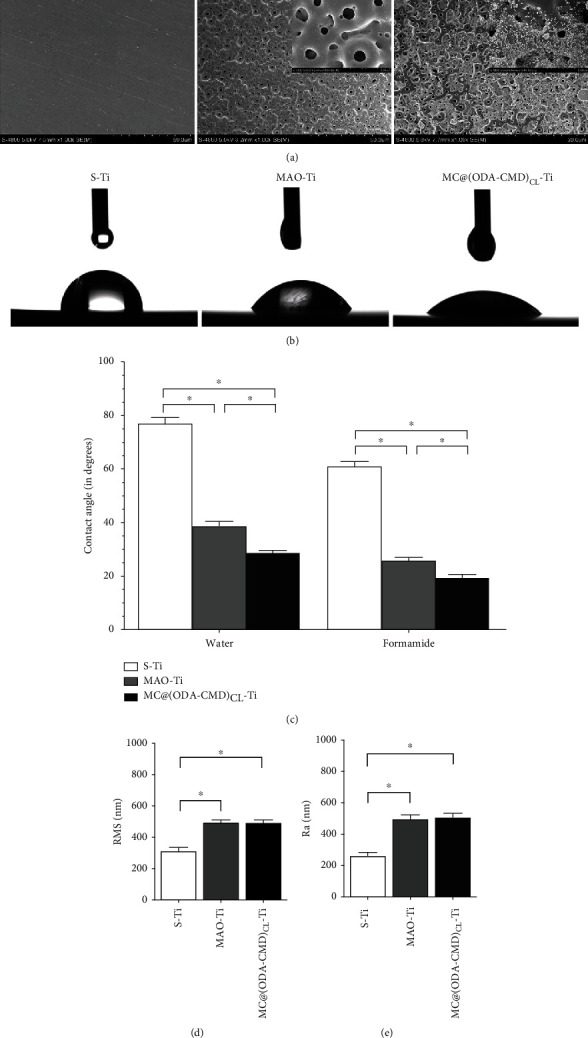
Surface characterization of the MC-loaded nanomicelle coating on titanium sheets. (a) SEM images of three types of titanium sheets (S-Ti, MAO-Ti, and MC@(ODA-CMD)_CL_-Ti) under low magnification (inset) under high magnification. (b) Images of contact angle measurement for S-Ti, MAO-Ti, and MC@(ODA-CMD)_CL_-Ti. (c) Statistical analysis of contact angle measurements. The data are presented as mean ± SD (*n* = 6, ^∗^*P* < 0.05). (d) Quantitative measurement of surface roughness of S-Ti, MAO-Ti, and MC@(ODA-CMD)_CL_-Ti by RMS value and (e) Ra value. Data are presented as mean ± SD (*n* = 6, ^∗^*P* < 0.05).

Representative SEM photographs of the three types of titanium sheets are shown in Figure 4(a). The S-Ti sheets were relatively smooth after being ground using silicon carbide paper. The titanium surface after MAO treatment showed a porous morphology with pores of 1-5 *μ*m, providing many footholds for drug-loaded nanomicelles. Further, the drug-loaded nanomicelles were loaded within the pores on the MAO-Ti surface after oscillation and cross-linking.

#### 3.3.2. Contact Angle

The hydrophilicity of different titanium surfaces was determined by contact angle measurements. The obtained droplets (water and formamide) on different titanium surfaces (Figure 4(b)) were quantitatively analyzed, in which S-Ti was found to have the highest contact angle compared with MAO-Ti and MC@(ODA-CMD)_CL_-Ti. After microarc-oxidized treatment, the contact angle degree was significantly decreased for MAO-Ti compared with S-Ti. Furthermore, when MAO-Ti was fabricated with a drug-loaded nanomicelle coating, the contact angle of MC@(ODA-CMD)_CL_-Ti was significantly lower than that of MAO-Ti (Figure 4(c)). Thus, these results suggest that MC@(ODA-CMD)_CL_-Ti had a better hydrophilic performance than S-Ti and MAO-Ti.

#### 3.3.3. Roughness

The quantitative analysis of surface roughness showed that the RMS and Ra values of S-Ti were 314.5 ± 61.6 nm and 264.5 ± 49.76 nm (Figures 4(d) and 4(e)). Also, microarc oxidation led to a significant increase in the RMS and Ra values. The corresponding RMS and Ra values of MAO-Ti were 497.5 ± 49.5 nm and 498.7 ± 60.63 nm, while the RMS and Ra values of MC@(ODA-CMD)_CL_-Ti were 495.7 ± 49.54 nm and 508.8 ± 61.4 nm. No significant differences were observed in the RMS and Ra values between MAO-Ti and MC@(ODA-CMD)_CL_-Ti. These results showed that MAO-Ti and MC@(ODA-CMD)_CL_-Ti shared a similar surface roughness, higher than that of S-Ti.

### 3.4. Drug Release Properties

**Figure 5:**
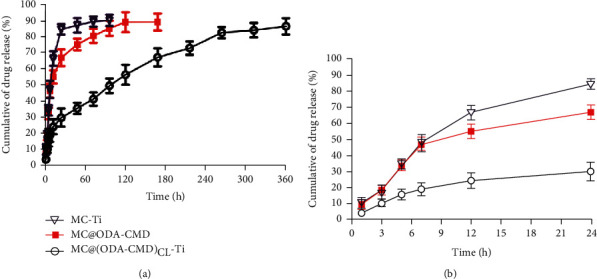
The cumulative drug release of MC from different specimens at various periods. (a) Cumulative drug release curves during 360 h. (b) Cumulative drug release curves of the first 24 h.

The cumulative release profile of MC during the 360 h release period is shown in Figure 5(a). When free MC was coated on the titanium sheet, the cumulative drug release reached 90.3 ± 3.5% after 96 h, while that of MC@ODA-CMD reached 89.3 ± 5.3% after 168 h. However, after MC@(ODA-CMD)_CL_ was coated on MAO-Ti, their sustained release times were prolonged, with the final release rate at 360 h being 86.6%. To better understand the initial rapid release, we extracted the data in the first 24 h of the release curve, developed in Figure 5(b). The cumulative release value of MC@(ODA-CMD)_CL_-Ti at 24 h was found to be 30 ± 5.7%, which was significantly lower than that of MC-Ti and MC@ODA-CMD. The cumulative release value of the former was 84.7 ± 3.2%, and the latter was 67.1 ± 5.2%.

### 3.5. Antibacterial Effects

From the above results, we could observe that the stability, drug loading capacity, and drug release performance of MC@(ODA-CMD)_CL_ were superior to MC@ODA-CMD. Thus, in the subsequent experiments, MC@ODA-CMD was excluded, and only MC@(ODA-CMD)_CL_ was tested.

#### 3.5.1. Antibacterial Rate (AR)

**Figure 6:**
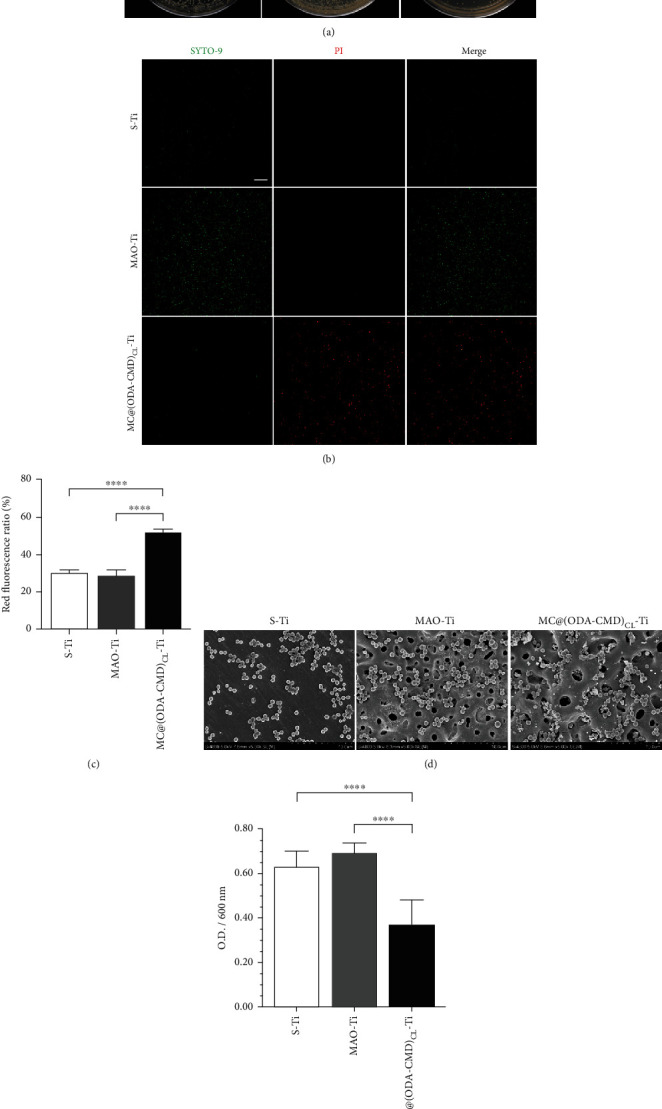
(a) Bacteria colonies cultivated on MHA plates of S-Ti, MAO-Ti, and MC@(ODA-CMD)_CL_-Ti. (b) Fluorescence staining showing the live/dead distribution of *S. aureus* on S-Ti, MAO-Ti, and MC@(ODA-CMD)_CL_-Ti (green for live bacteria and red for dead bacteria); scale bars = 50 *μ*m. (c) Quantification results of live/dead immunofluorescence staining (*n* = 5, ^∗∗∗∗^*P* < 0.0001). (d) SEM showing S. aureus colonies on S-Ti, MAO-Ti, and MC@(ODA-CMD_)CL_-Ti. (e) Statistical analysis of antibacterial activities of three types of titanium sheets (CCK-8 assay). The data are presented as mean ± SD (*n* = 6, ^∗∗∗∗^*P* < 0.0001).

Here, bacteria colony counting was performed to quantitatively analyze the antibacterial effect of the specimens, including planktonic and immobilized bacteria. From the photographs of the bacteria colonies on Mueller-Hinton agar (MHA) plates, we found that the number of bacterial colonies on MC@(ODA-CMD)_CL_-Ti was lowest compared with S-Ti and MAO-Ti (Figure 6(a)). Quantitative analysis showed that the average AR of MC@(ODA-CMD)_CL_-Ti specimens was 80.6%, while that of MAO-Ti was -443.6%.

#### 3.5.2. Bacterial Viability and Morphology

As shown in Figure 6(b), most bacteria that adhered to S-Ti and MAO-Ti were stained fluorescent green after 24 h of incubation, indicating they were alive and had intact membranes. In contrast, almost all bacteria attached to MC@(ODA-CMD)_CL_-Ti were stained fluorescent red, suggesting that they were dead and their membranes were damaged. Quantification of immunofluorescence was performed to assess the percentage of dead bacteria, demonstrating a red fluorescence ratio of 30% on S-Ti, 28% on MAO-Ti, and 52% on MC@(ODA-CMD)_CL_-Ti (Figure 6(c)). Further, SEM images showed lower levels of bacteria colonies persisted on MC@(ODA-CMD)_CL_-Ti compared with S-Ti and MAO-Ti (Figure 6(d)), with some of the bacteria in contact with the drug-loaded nanomicelles exhibiting deformed membranes.

#### 3.5.3. CCK-8 Assay

Here, the CCK-8 assay was used to determine the viability of S. aureus on the three types of titanium specimens after 24 h. Similar to the results of fluorescence staining, the OD value of MC@(ODA-CMD)_CL_-Ti was found to be significantly lower than that of S-Ti and MAO-Ti, suggesting the viability of S. aureus on MC@(ODA-CMD)_CL_-Ti. No significant differences were found between the OD values of the S-Ti and MAO-Ti groups. These results showed that after 24 h, the viability of S. aureus on MC@(ODA-CMD)_CL_-Ti was significantly decreased compared with that on S-Ti and MAO-Ti (Figure 6(e)).

### 3.6. Cell Behavior on Modified Titanium Specimens

#### 3.6.1. Viability of Human Skin Fibroblasts

**Figure 7:**
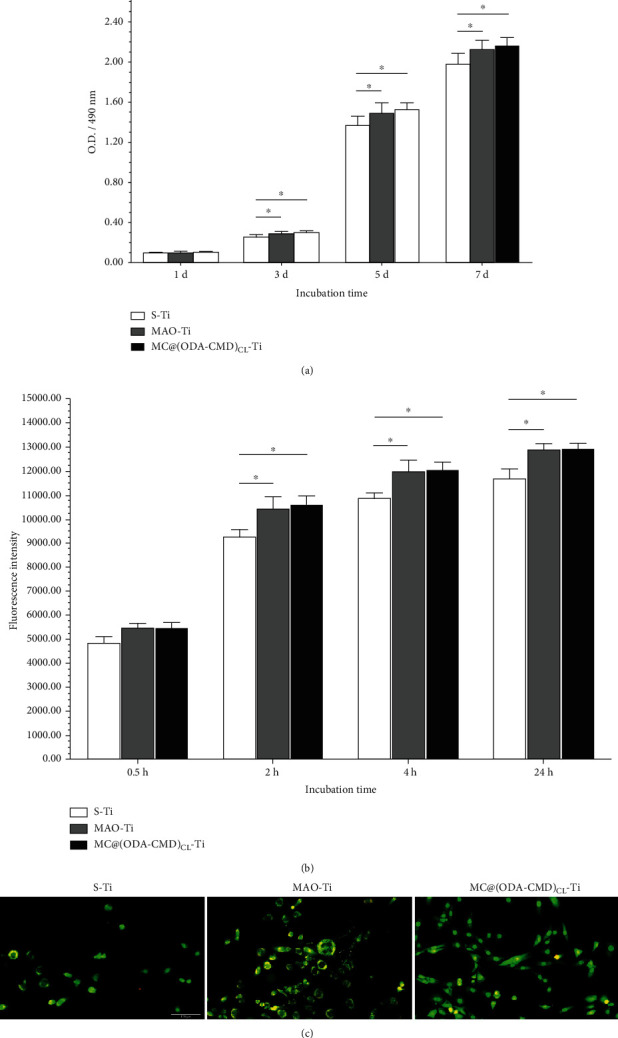
(a) Cell viability on S-Ti, MAO-Ti, and MC@(ODA-CMD)_CL_-Ti (CCK-8 assay). The data are presented as mean ± SD (*n* = 6, ^∗^*P* < 0.05). (b) Quantitative analysis of cell adhesion attached to three types of titanium sheets. The data are presented as mean ± SD (*n* = 6, ^∗^*P* < 0.05). (c) Observation of human skin fibroblasts via AO/EB fluorescence staining after incubation for 24 h on three types of titanium sheets. Scale bar = 100 *μ*m.

The viability of human skin fibroblasts on different specimens was assessed with CCK-8 assays during the 7-day period (Figure 7(a)). On days 3, 5, and 7, the viability of fibroblasts grown on both MAO-Ti and MC@(ODA-CMD)_CL_-Ti was found to be significantly higher than that of S-Ti. Further, the cell viability continued to increase from days 1 to 7, indicating that the three types of titanium specimens possessed good biocompatibility.

#### 3.6.2. Adhesion of Human Skin Fibroblasts

As shown in Figure 7(b), cell adhesion to MC@(ODA-CMD)_CL_-Ti and MAO-Ti increased significantly compared to the S-Ti groups at 2, 4, and 24 h. However, there was no significant difference in fibroblast adhesion between MAO-Ti and MC@(ODA-CMD)_CL_-Ti at these time points.

#### 3.6.3. AO/EB Fluorescence Staining

When the fibroblasts were observed under an inverted fluorescent microscope, the cells grown on S-Ti and MAO-Ti were found to be spherically shaped, with poorly stretched morphology, narrow extensions, and few extended pseudopods. In contrast, the fibroblasts on the MC@(ODA-CMD)_CL_-Ti were well-flattened with clear lamellipodia and filopodia projecting from the cell periphery. More cells on MAO-Ti and MC@(ODA-CMD)_CL_ were observed compared to those of the S-Ti group (Figure 7(c)).

## 4. Discussion

Peri-implantitis around percutaneous titanium implants mainly occurs due to initial bacterial adhesion and biofilm formation [10]. Once bacteria have invaded and destroyed the seal between the skin and implant, the osseointegration of the percutaneous implant loses its barrier protection, resulting in bone resorption or even implant failure [11]. Thus, many scholars have adopted various strategies to construct antibacterial coatings on the surface of implants' titanium to prevent bacterial infection. However, instability, insufficient drug release time, and potential cytotoxicity were among the limitations associated with these reported constructs [33–35]. To improve the coating design, in this study, we developed shell cross-linked amphiphilic nanomicelles (ODA-CMD)_CL_ as a drug carrier, in which antibiotics (MC) were loaded into its core and loaded into the micropores of MAO titanium surfaces to form an antibacterial coating. Then, we conducted a series of experiments to evaluate the potential efficacy of the drug-loaded nanomicelles and investigate the drug release properties, antibacterial ability, and biocompatibility of the coating.

Nanomicelles based on dextran and its derivatives have been developed for drug delivery systems in medical applications due to their biocompatibility and bioavailability [36]. However, the existence of *α*-1, 6 glycosidic bonds in dextran provides an increase in chain mobility, contributing to their high solubility in many solvents and reducing their stability of nanomicelles [25, 37], which limits their long-term application as a drug carrier. In this regard, shell cross-linking is an easy and effective alternative to enhance the stability of nanomicelles because it can produce covalently stabilized structures to prevent the dissociation of nanomicelles and regulate drug release [38, 39]. In this study, we found that the CMC of (ODA-CMD)_CL_ was significantly lower than that of ODA-CMD, suggesting that (ODA-CMD_)CL_ was more stable than ODA-CMD, which were validated in stability assays.

The properties of biomaterials, such as surface roughness and hydrophilicity, can greatly influence bacterial adhesion and biological behaviors. Previous studies have shown that high surface roughness can enhance bacterial adhesion and the formation of bacterial biofilms, while an increase in surface hydrophilicity can facilitate cell adhesion, spreading, and proliferation, thus improving the biocompatibility of biomaterials. In this study, we found that the addition of MC@(ODA-CMD)_CL_ into MAO coating did not affect its surface roughness but significantly increased the hydrophilicity of MC@(ODA-CMD)_CL_-Ti compared with that of MAO-Ti. This increase in surface hydrophilicity may be explained by the fact that CMD has a high density of hydroxyl groups, which could thus increase the hydrophilicity of the coating [40].

Sustained drug release can maintain the inhibitory concentration for a certain time in the postoperative peak infection period. Several studies have attempted to use various methods for sustained drug release. MC on the modified titanium surface with graphene oxide exhibited a slow-release behavior for 168 h and showed an excellent antibacterial effect [41]. Dextran-based amphiphilic polymers were used for the sustained release of rapamycin for 7 days previous study [42]. In this present study, MC@ODA-CMD provided a rapid initial release with a cumulative release rate of about 67% within the first 24 h, followed by a sustained drug release with a cumulative release rate of 89.3% after 168 h. After MC@(ODA-CMD)_CL_ was coated on the MAO-Ti surface, only a cumulative release rate of 30% was observed in the first 24 h, and the sustained release time was extended to 360 h with a cumulative release rate of 86.6%. This increase in sustained drug release time could be attributed to the shell cross-linking, which led to a stabilization of the nanomicelles and hydrophobic interactions between MC and ODA. Additionally, changing the size of nanomicelles and MAO coating thickness may affect the drug release profile. In this regard, further studies are warranted to optimize the size of nanomicelles and the thickness of MAO coatings.

Two main challenges that titanium implants face in terms of long-term success in *in vivo* settings are biomaterial-related infections and compromised tissue integration [43]. Their relationship was previously described as a “race for surface” by Gristina, indicating that bacteria adhesion and host cell integration compete to reach the implant's surface [13]. In this context, reducing the number of bacteria or restricting the adhesion of bacteria to the implant surface could limit subsequent biofilm formation and facilitate host cell attachment, leading to favorable tissue integration. Our results showed that the fabrication of MC@(ODA-CMD)_CL_ with MAO titanium significantly outperformed S-Ti and MAO-Ti in inhibiting the adhesion and proliferation of S. aureus. The antibacterial effect of the coating was mainly due to the release killing by the nanomicelles incorporated in the titanium pores of MAO. When MCs were released from the nanomicelles, they entered the bacterial cells and inhibited bacterial protein synthesis [44]. In this study, we observed that MAO-treated titanium significantly promoted the adhesion and proliferation of S. aureus, which concurs with the findings of a previous study [45]. However, in another study, the authors reported that MAO-treated titanium could only partly restrain bacteria adhesion and growth [46]. We hypothesize that this discrepancy could be related to the different electrolytes used during the MAO process in these studies because the antibacterial ability of MAO titanium is not only related to its surface structure but also its chemical composition.

Meanwhile, the fundamental requirement of coating technology is that it should be biocompatible for further clinical application. Subcutaneous implantation studies and tissue response tests have confirmed the biocompatibility of dextran-based delivery systems, indicating that dextran is not associated with immunological reactions or toxicity to human cells [47, 48]. Similar observations were found in this study because we found that ODA-CMD and (ODA-CMD)_CL_ did not possess cytotoxicity against human cells. Fibroblasts are the main type of connective tissue cells around implants. They play a crucial role in soft tissue integration to protect implants from the outer bacterial environment by initiating cellular signaling processes and secreting extracellular matrix [49]. In this study, we further examined the effects of S-Ti, MAO-Ti, and MC@(ODA-CMD)_CL_-Ti on fibroblasts' viability, adhesion, and morphology to evaluate their biocompatibility. Our results showed that the fibroblasts seeded on MAO-Ti and MC@(ODA-CMD)_CL_-Ti demonstrated better cell viability and adhesion than those seeded on S-Ti. In addition, we also observed that the viability and adhesion of fibroblasts seeded on MC@(ODA-CMD)_CL_-Ti grew continuously throughout the observation period. Subsequently, morphology assays performed on fibroblasts on MC@(ODA-CMD)_CL_-Ti showed that the fibroblasts on MC@(ODA-CMD)_CL_-Ti exhibited better spreading characteristics with lamellipodia and filopodia projecting from their cell periphery. Previous studies showed that high surface hydrophilicity could promote cell adhesion, viability, and other biological behaviors [50], which could therefore explain the enhanced biocompatibility of MC@(ODA-CMD)_CL_-Ti.

The coatings proposed in this study exhibited good drug release capability, antibacterial activity, and biocompatibility. Further, the obtained results demonstrated promising experimental evidence for further using nanomicelles on titanium surface coatings. In our future studies, we would focus on the antibacterial effects of more bacteria and *in vivo* experiments to further explore the capabilities of nanomicelles.

## 5. Conclusions

In this study, we reported our findings on a new type of nanomicelle as a drug carrier for antibacterial coatings on MAO titanium surface. The proposed strategy demonstrated promising efficacy as a potential solution to prevent bacterial infections in percutaneous implants. Using the present findings as reference, further studies could be initiated to explore different strategies, such as nanomicelle antibacterial coatings that can smartly respond to microenvironments (pH, enzyme, etc.) or exogenous stimuli (light, temperature, etc.) and injectable nanomicelle hydrogels, for enhancing the properties of nanomicelles.

## Figures and Tables

**Table 1:** Characteristics of drug-loaded nanomicelles (*n* = 3, $\bar{x}\pm s$).

Nanomicelles	Size (nm)	PDI	Zeta (mV)	DL (%)	EE (%)	CMC (mg/L)
MC@ODA-CMD	118 ± 10	0.21 ± 0.03	−18.6 ± 1.9	15.67 ± 0.2	43.84 ± 5.3	4.9
MC@(ODA-CMD)_CL_	130 ± 16	0.19 ± 0.08	−14.3 ± 2.3	17.31 ± 0.8	50.18 ± 3.8	3.1^∗^

## Data Availability

The data used to support the findings of this study are available upon request from the corresponding authors.

## Supplementary Materials

Supplementary Materials Schematic illustration about the MC@(ODA-CMD)_CL_-Ti coating and its properties.
