# Supplementary material for: Carboxymethyl Dextran-Based Nanomicelle Coatings on Microarc Oxidized Titanium Surface for Percutaneous Implants: Drug Release, Antibacterial Properties, and Biocompatibility
Source: Biomed Res Int. 2022 Jul 12;2022:9225647. doi: 10.1155/2022/9225647 (PMC9296324; doi:10.1155/2022/9225647)
Supplement: Supplementary Materials — Schematic illustration about the MC@(ODA-CMD)CL-Ti coating and its properties. [file 9225647.f1.docx]

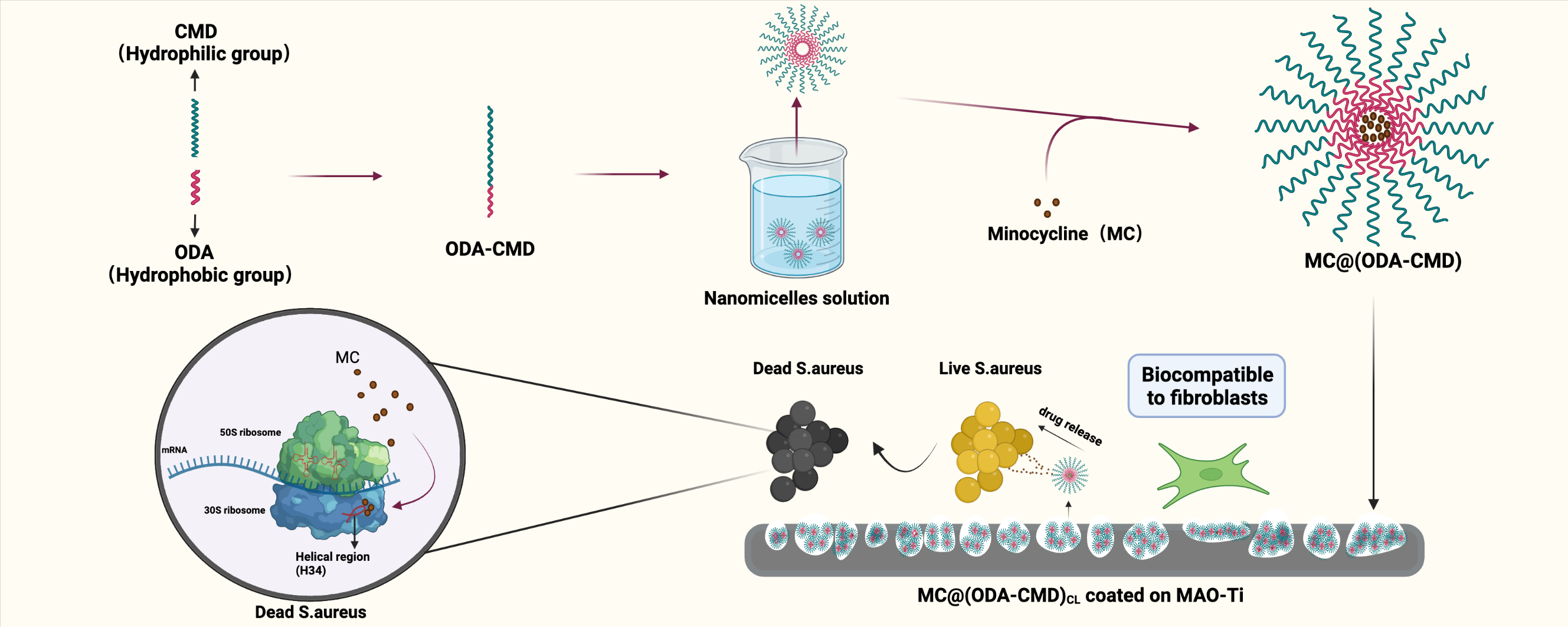


Supplementary Figure 1: Schematic illustration about the preparation of MC@(ODA-CMD)_CL_-Ti coating and its antibacterial properties and biocompatibility.
